# A synthetic microbial Daisyworld: planetary regulation in the test tube

**DOI:** 10.1098/rsif.2023.0585

**Published:** 2024-02-07

**Authors:** Victor Maull, Jordi Pla Mauri, Nuria Conde Pueyo, Ricard Solé

**Affiliations:** ^1^ Institució Catalana de Recerca i Estudis Avançats, Psg Lluis Companys, Barcelona, Spain; ^2^ Complex Systems Lab, Universitat Pompeu Fabra, Barcelona 08003, Spain; ^3^ EMBL Barcelona, European Molecular Biology Laboratory (EMBL), Barcelona 08003, Spain; ^4^ Santa Fe Institute, 1399 Hyde Park Road, Santa Fe, NM 87501, USA

**Keywords:** Daisyworld, homeostasis, Earth systems science, synthetic biology, terraformation

## Abstract

The idea that the Earth system self-regulates in a habitable state was proposed in the 1970s by James Lovelock, who conjectured that life plays a self-regulatory role on a planetary-level scale. A formal approach to such hypothesis was presented afterwards under a toy model known as the Daisyworld. The model showed how such life-geosphere homeostasis was an emergent property of the system, where two species with different properties adjusted their populations to the changing external environment. So far, this ideal world exists only as a mathematical or computational construct, but it would be desirable to have a real, biological implementation of Lovelock’s picture beyond our one biosphere. Inspired by the exploration of synthetic ecosystems using genetic engineering and recent cell factory designs, here we propose a possible implementation for a microbial Daisyworld. This includes: (i) an explicit proposal for an engineered design of a two-strain consortia, using pH as the external, abiotic control parameter and (ii) several theoretical and computational case studies including two, three and multiple species assemblies. The special alternative implementations and their implications in other synthetic biology scenarios, including ecosystem engineering, are outlined.

## 1. Introduction

Our biosphere is the result of a long-term evolutionary experiment where living life forms and their environments have been interacting closely and on multiple scales through millions of years. The composition of our biotas has been changing, sometimes in dramatic ways, as shown by the fossil record of life [1,2]. This evolutionary process has been taking place on a planet that has also experienced profound changes. Some were caused (or driven) by astronomical phenomena, from deterministic orbital cycles to fateful asteroid impacts. However, major changes took place as a consequence of the entangled nature between climate and the biosphere [3]. The deep connections between environment and life leave a mark in the geological past. How has life influenced climate and vice versa? As pointed out by Vernadsky [4], the emergence of life fundamentally transformed the geosphere. One specially interesting observation made by geochemist James Lovelock in the 1970s was the realization that our planet should have been driven by our Sun into higher temperature regimes.

We know from our two closest planetary neighbours, Mars and Venus, that steady changes in physical parameters can trigger runaway effects leading to an inhabitable planet [5]. In general, positive feedback loops can drive a planet to extreme steady states, from boiling temperatures to a snowball [3,6]. And yet, life on Earth has emerged and diversified, somehow dealing with the impacts of external drivers. To explain such stability, Lovelock suggested that an active coupling between life and physical systems make up the planet [7–10]. To make this point more explicit, Watson & Lovelock proposed in 1983 a simple model of planetary regulation known as the Daisyworld model (DWM) [11,12]. In a nutshell, the DWM considered an ideal planet where two kinds of agents, namely black and white daisies, had a distinct impact on planet albedo thus changing the local temperature in a way that could allow for climate stability over a wide range of solar luminosity values. Since its formulation, the DWM has become a canonical model for Earth system science [13] and has been used to explore a very diverse range of problems, from tipping points [14,15], to evolutionary dynamics [16,17] to climate bistability in exoplanets [18]. In contrast to other problems, the planetary scale makes it rather unlikely to approach this coupling between environment and ecology in terms of controlled experimental conditions. Within synthetic biology, successful modelling and implementation of engineered ecosystems has been achieved, including cooperative consortia [19,20], predator–prey systems [21] or even multispecies assemblies [22–24]. Could such a synthetic counterpart be found for the DWM?

A general formulation of the Watson–Lovelock model is described by means of a set of coupled differential equations defining an ecological model, namely (see [25] and references therein):

1.1 $$\frac{dx_{k}}{dt}=x_{k}\left(\beta_{k}(T)\left[1-\sum_{\;j=1}^{n}x_{\;j}\right]-\delta_{k}\right)$$

where *x*_*k*_ (*k* = 1, …, *n*) is the population size of the *k*th species. The parameter *β*_*k*_(*T*) is a temperature-dependent growth rate, whereas *δ*_*k*_ is the corresponding decay (death) rate. A logistic saturation is introduced by the $1-\sum_{\;j}x_{\;j}$ term.

**Figure 1.**
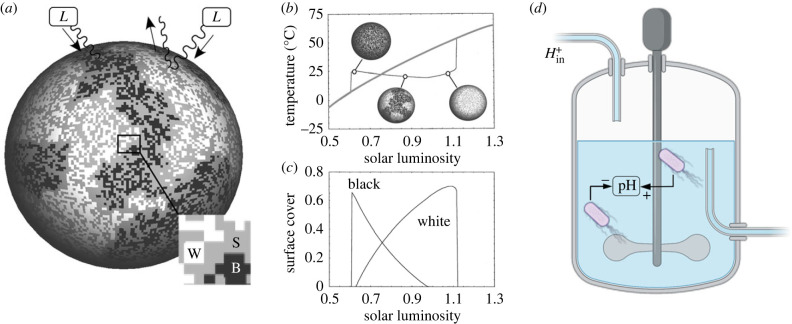
The conceptual feedbacks in the Watson–Lovelock model, which is the canonical formulation of the DWM. Using a two-dimensional surface (*a*), increasing levels of solar luminosity *L* trigger the growth (after a threshold) of two populations of plants, indicated as *B* and *W* (white and black squares, grey squares stand for bare soil *S*, see inset on the left bottom, using a zoom on the area indicated). These are identified as black and white daisies, respectively, in the DWM. Opposite feedbacks emerge from the effects of albedo by the two kinds of daisies. As a consequence of these nonlinear couplings, as shown in (*b*), the planet temperature can be stabilized (instead of just simply growing with *L*, grey line) for a wide range of *L* values, thus indicating a homeostatic response due too the biosphere–climate system. Such stabilization is obtained by means of population arrangements between W and B states (*c*). In this paper, an equivalent system is proposed (*d*) using a bioreactor where an external input is also present ($H_{\mathrm{in}}^{+}$) that would increase the pH of the medium, unless feedback controls are present. In this case, two different strains of bacteria able to increase or decrease the pH would replace the daisies.

The basic feedback loops involved are represented in figure 1*a* for the standard two species (*n* = 2) model. Increasing luminosity can trigger the growth of both kinds of vegetation, at rates indicated by *β*_*k*_(*T*) (with *k* = 1, 2), here using *k* to refer to the different kinds of daisies, as a generalization, further on we specify which daisies are at play. In the best known (and simpler) version, two species are considered and the following model was explored, for a ‘world’ involving black (*x*_*b*_) and white (*x*_*w*_) daisies

1.2 $$\frac{dx_{w}}{dt}=x_{w}(\beta_{w}(T)[1-x_{w}-x_{b}]-\delta_{w})$$

and

1.3 $$\frac{dx_{b}}{dt}=x_{b}(\beta_{b}(T)[1-x_{w}-x_{b}]-\delta_{b})$$

where [1 − *x*_*w*_ − *x*_*b*_] introduces the available bare soil that both species can occupy. In figure 1*a*, we sketch the basic model components using a spatial version [26–28], with dark, white and grey sites indicating black and white daisies and bare soil, respectively (lower inset, figure 1*a*). These equations remind us of a competition model, but the relevant complexity is captured within the *β*_*i*_(*T*) factors (with *i* = *b*, *w*), for black and white, defined by means of a single-humped function involving an optimum. The details of this model can be found elsewhere [11,12] but the crucial intuition is that the growth rates of each kind of daisy is affected by temperature in opposite ways. At low temperature, black ones will thrive since they will warm their local environment and spread. As *T* increases, white daisies are favoured because they cool their environment locally. The area covered by each species contributes to the albedo and affect local temperatures in such a way that a global temperature regulation emerges, leading to a stable regime of local temperatures, as shown in figure 1*b*. Here the grey, increasing trend indicates how temperatures will rise in the absence of biological control. The underlying populations of each type are also shown in figure 1*c*.

The DWM, despite its specific traits and potential challenges in translating it to the real biosphere, offers a valuable principle of living homeostasis that warrants consideration under broader assumptions. The concept of a living ecosystem that can respond to external forces and readjust itself to preserve diversity or some global property is inherently intriguing. It opens the door to creating mathematical and computational models representing such homeostatic ecologies, but an even more relevant prospect lies in constructing a real living ecosystem that echoes Lovelock’s vision. Synthetic biology emerges as an ideal candidate for achieving this ambitious goal. Through genetic engineering techniques, scientists have successfully designed cells with novel functionalities and orchestrated their interactions in intricate ways. Consequently, the main objective of this paper is to demonstrate the theoretical feasibility of constructing a synthetic Daisyworld.

Creating a living surrogate of the original DWM could help explore the general problem in novel ways, as well as providing a rich context to explore the role of lower-scale features (such as molecular regulation) on the global regulation processes. The challenge is not minor: an experimental surrogate of the planetary coupling between life and environment involving cooperative feedback is far from obvious. However, although a potential choice could involve using temperature as the driving parameter, there are other no less important properties that have also been controlled at the planetary level. One of them is acidity [9]: despite the tendency towards acidification associated with increasing oxidation of the atmosphere, the mean pH of the oceans has been remarkably stable over the Phanerozoic [29–32]. As pointed out in [3], *'the daisies are a surrogate for any kind of life that can affect the global temperature—and temperature could equally well be any other environmental variable that life cares about, for instance the oxygen concentrations, or pH."* And indeed in their early analysis of the problem, Margulis & Lovelock already pointed out that, along with temperature and atmosphere composition, ocean acidity has been under feedback control [9].

In this work, we choose pH as a relevant environmental parameter that can be used as an external, tuneable input. Thus, we instead consider an alternative that allows a straightforward approach that captures all the relevant feedbacks and allows for a microcosm/mesocosm implementation (figure 1*d*) based on a synthetic microbial ecosystem where pH is tuned by two populations that will play the role of our daisies.

## 2. Methods

This paper delves into two crucial aspects concerning the definition of a synthetic microbial Daisyworld. Firstly, we explore a collection of genetic circuits linked to a two-strain consortia specially engineered to regulate the environmental pH. Drawing inspiration from recent research on the controlled management of industrial fermentation [33], we present a novel synthetic microbial consortium aligned with the regulatory feedback principles of the DWM. Secondly, we demonstrate how the well-established impacts of acidity on microbial growth can be mapped onto ecological network models, akin to those of the DWM based on temperature and albedo.

### 2.1. Microbial Daisyworld: synthetic circuits

**Figure 2.**
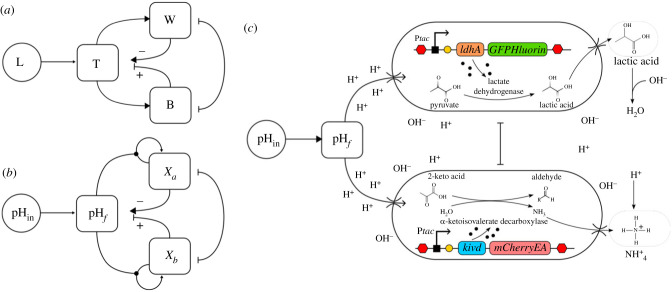
The synthetic microbial Daisyworld. The logic of the zero-dimensional (non-spatial) DWM is summarized in (*a*) in terms of the interactions between planetary temperature and the distinct role played by the two kinds of daisies (B and W). Using our framework, where acidity would be the controlled variable. In this context, the acidity level within the medium influences cellular replication (self-replication loops). Simultaneously, both species reciprocally influence the acidity of the medium. A similar logic of feedback loops can be described (*b*) where now two microbial populations would also reduce or increase local acidity. The whole design, including the corresponding genetic constructs, is depicted in (*c*).

The success of implementing a pH-based synthetic DWM hinges on two key factors: (i) ensuring a strong alignment with the growth response assumptions originally formulated by Lovelock, which were based on local temperature and (ii) skilfully engineering microbial–environment interactions to effectively regulate local pH, simulating the feedback mechanisms depicted in figure 2*a*. Extensive research has been conducted on the growth responses of microorganisms to pH, leading to the development of various mathematical models to characterize their behaviour. Notably, the analysis of the relative growth rate against pH reveals a distinctive inverted parabolic pattern [34], which harmonizes with the foundational assumptions of the DWM. These assumptions entailed a smooth curve with a single optimum and well-defined limits. Empirical data from diverse species, such as *Escherichia coli* or *Listeria* sp., demonstrate symmetric functional responses within a pH range of [pH_*m*_, pH_*M*_], where pH_*m*_ and pH_*M*_ represent the zero-growth limits of the fitted curve [34].

While different mathematical models, such as the Presser [35] or Lambert–Pearson [34,36] models, have been proposed, they all exhibit a near-parabolic behaviour. Consequently, the effects of pH closely align, in mathematical terms, with the control space assumptions of the DWM. This congruence underscores the potential feasibility of establishing a synthetic microbial Daisyworld based on pH regulation.

Is it possible to design a synthetic consortium comprising two strains that can effectively control the acidity of the environment? Our aim is to create a pair of designed strains capable of responding to changes in pH in a manner that mirrors Lovelock’s concept, as illustrated in figure 2*a*,*b*. In this scenario, the two engineered strains would function in opposing ways: although they share a common optimum pH, they would either increase or decrease the environmental acidity/alkalinity levels. This complementary behaviour would lead to a mutual self-regulation, ideally maintaining a constant environmental pH value. In a broader context, this approach represents a specific application of metabolic engineering facilitated by synthetic biology [37]. Unlike traditional methods that involve continuous monitoring and manual addition of sterile bases and acids as needed [33,38], our case study focuses on developing a system capable of self-correcting deviations from the optimal pH. By doing so, we aim to eliminate the need for constant fine-tuning, making the process more efficient.

We propose the utilization of two engineered cell types (figure 2*c*): the acid-producing and the base-producing strains growing in a chemostat supplying a constant flow of nutrients and removing a constant proportion of cells. The acid-producing strain incorporates the *ldhA* functional gene responsible for expressing lactate dehydrogenase. This enzyme facilitates the conversion of pyruvate to lactic acid, resulting in a decrease in external pH [33]. Conversely, the base-producing strain employs the *kivd* functional gene, encoding *α*-ketoisovalerate decarboxylase. This enzyme is involved in the decarboxylation of branched-chain *α*-keto acids derived from branched-chain amino acids transamination into aldehydes [39]. In simpler terms, it catalyses the conversion of 2-keto-acid to aldehyde, leading to an overproduction of ammonia, subsequently reducing the medium’s acidity by forming ammonium ions. While other genes like *glsA* or *gadA* could have been considered to catalyse ammonium ion production through alternative processes [33], using them might result in lower yields.

To ensure robustness in the experiments, we propose employing the *E. coli* knockout strain with a deletion in the glutamine synthetase gene (Keio collection JW3841-1), yielding the Δ*glnA* genotype. By blocking ammonia re-uptake through this deletion, we can enhance the performance of the base-producing strain. Additionally, we recommend a deletion in Δ*lldP*, which encodes an inner membrane permease involved in lactate uptake [40,41]. This modification further supports the function of the acid-producing strain, ensuring its efficiency in the system.

Regarding promoter usage, we propose employing *pTac* in both genetic devices, as it is commonly used to control and overexpress recombinant proteins. However, any controllable promoter without leakiness and with high fold change expression could also be suitable. If an imbalance is detected in the production of both *ldhA* and *kivd* genes, the genetic devices could be easily modified to address the issue by changing one device to a different controllable promoter, such as *pTet-ldhA* or *pTet-kivd*. Our proposed model involves the production of strong acids/bases, but the generation of such highly reactive chemicals would be detrimental to the survival of the bacteria. Therefore, we suggest using an experimental system with production of weaker, autonomously controlled reactants. This approach aims to minimize differences between the model and the experiments.

Furthermore, to facilitate real-time dynamics of the two strains and track the experimental process, fluorescent reporter genes would be used. We suggest using pH-sensitive fluorophores such as green and red fluorescent proteins. The green fluorescent protein *GFP-pHluorin* and the red fluorescent protein *mCherryEA* have been identified as ratiometric pH sensors, where the protonation of the chromophore is pH-dependent. They exhibit an approximately eightfold increase in expression with increasing pH values, ranging from 5 to 9 [42,43].

This synthetic consortium would grow in a bioreactor environment (figure 1*c*) with a stable supply of fresh media ensuring nitrogen availability (required for a proper ammonia production).

### 2.2. Microbial Daisyworld: two-species model

Using the known parabolic shape of relative growth responses to acidity, a simple mathematical model can be build under the assumption that the two synthetic strains described above can be engineered. The model now requires taking into account the fact that changes can be observed on two, connected scales. One is described in terms of relative cell populations. The second is a cell-level dynamics associated with the pH balances between the intracellular concentration and the one they perceive from their local environment.

The equations that are used to describe the cell dynamics are a coupled set of equations describing the growth of each cell type, similar to standard replicator equations from population genetics:

2.1 $$\frac{dX_{a}}{dt}=X_{a}[\phi (X)\beta ({\mathrm{pH}}_{a})-\delta ]$$

2.2 $$\frac{dX_{b}}{dt}=X_{b}[\phi (X)\beta ({\mathrm{pH}}_{b})-\delta ]$$

where *X*_*a*_ is the concentration of acid-producing cells, *X*_*b*_ is the concentration of base-producing cells, $\phi (X)=1-(X_{a}+X_{b})$ describes the negative feedback associated with the finite amount of available space for cells to occupy and *δ* is the dilution rate. The functional form of *β* describing the change in growth rate depending on the perceived pH is here chosen as

2.3 $$\beta ({\mathrm{pH}}_{x})=1-\frac{{\left({\mathrm{pH}}_{\mathrm{opt}}-{\mathrm{pH}}_{x}\right)}^{2}}{{\left({\mathrm{pH}}_{\mathrm{opt}}\pm {\mathrm{pH}}_{\lim}\right)}^{2}}$$

(other choices gave similar results, provided that the dependence is one-humped). It describes a symmetric, single-peaked function with its maximum output located at pH_*x*_ = pH_opt_, and positive output for an input in the range pH_*x*_ ∈ (pH_opt_ ± pH_lim_). It guarantees that all factors affecting cell growth that are pH-dependent, including the availability of nutrients, decreasing as pH strays from the optimum. The perceived pH depends on the level of acidity/alkalinity that each cell experiences on a local scale. Thus, we have:

2.4 $${\mathrm{pH}}_{a}={\mathrm{pH}}_{\;f}+\omega a_{e}\;\;{\mathrm{pH}}_{b}={\mathrm{pH}}_{\;f}-\omega b_{e}\;$$

where both *a*_*e*_ and *b*_*e*_ stand for the acid or base produced at the individual cell level and require specific dynamical assumptions (see below), and *ω* is the sensitivity to the produced substance. pH_*f*_ is described as the free pH in the media, that depends on the external input pH_in_ and the action of each population. We only take into account the external pH_*f*_ instead of the usual ΔpH as it is assumed that the cell’s internal pH will remain almost constant [44].

2.5 $$\frac{d{\mathrm{pH}}_{\;f}}{dt}={\mathrm{pH}}_{\mathrm{in}}+X_{a}a_{e}-X_{b}b_{e}-\delta {\mathrm{pH}}_{\;f}$$

Here, *a*_*e*_ and *b*_*e*_ stand for the external level of acidity/alkalinity a cell is able to excrete to the external surrounding media (see below). Furthermore, if we (reasonable) assume that the pH_*f*_ dynamics are fast, and thus we can use dpH_*f*_/d*t* ≈ 0 and in such a scenario we have:

2.6 $${\mathrm{pH}}_{f}={\mathrm{pH}}_{\mathrm{in}}+X_{a}a_{e}-X_{b}b_{e}$$

Additionally, we need to take in account the microscopic balances at the level of individual cells. Therefore, at the molecular level we can assume stoichiometry ruling the internal levels of acid or base production of each cell and the extracelular transport rates. We have the following reactions.

2.7 $$\emptyset \;\overset{\gamma }{\to }\;a_{i}\;\overset{k_{1}}{\underset{k_{2}}{⇌}}\;a_{e}\;\overset{\delta }{\to }\;\emptyset \;\;\emptyset \;\overset{\gamma }{\to }\;b_{i}\;\overset{k_{3}}{\underset{k_{4}}{⇌}}\;b_{e}\;\overset{\delta }{\to }\;\emptyset$$

Here, as has been previously pointed, *a*_*e*_ and *b*_*e*_ stand as the external concentration of acid or base in each type of cell, and *a*_*i*_ and *b*_*i*_ are the amount of acid or base that the cell produces inside the membrane. The production rate is *γ*. The interchange ratios between the inner cell and the exterior are *k*. The differential equations according to the reactions are

2.8 $$\frac{da_{i}}{dt}=\gamma -k_{1}a_{i}+k_{2}a_{e}$$

2.9 $$\frac{da_{e}}{dt}=k_{1}a_{i}-k_{2}a_{e}-\delta a_{e}$$

2.10 $$\frac{db_{i}}{dt}=\gamma -k_{e}b_{i}+k_{4}b_{e}$$

2.11 $$\frac{db_{e}}{dt}=k_{3}b_{i}-k_{4}b_{e}-\delta b_{e}$$

It can be assumed that those reactions happening at the molecular level, in fact, are much faster than the population dynamics, therefore, considering dΓ/dt=0 for each variable $\Gamma \in \{a_{i},b_{i},a_{e},b_{e}\}$. In this case, the equilibrium points can be described as constants.

2.12 $$a_{i}^{*}=\frac{\gamma (k-\delta )}{\delta k}\;\;a_{e}^{*}=\frac{\gamma }{\delta }$$

2.13 $$b_{i}^{*}=\frac{\gamma (k-\delta )}{\delta k}\;\;b_{e}^{*}=\frac{\gamma }{\delta }$$

Using the fast-relaxation assumption, we can now proceed to the analysis of the system dynamical patterns.

## 3. Results

Using the previous mathematical model approach and the assumptions made, it is possible now to study the expected dynamical behaviour of our proposed microbial Daisyworld. We first consider the two-species scenario (*A*) where a consortium of two microorganisms acts on pH and secondly the more general scenario where (*B*) a parasitic species is added and (*C*) multiple species are introduced. For low-dimensional (two to three species) cases, the system dynamics are computed numerically through an iterative Euler method, ensuring stability for both species and pH_*f*_ under each external pH input. The multispecies equations were numerically solved using a Runge–Kutta fourth-order approximation. In table 1, all the parameters and functions used in our models are summarized.

### 3.1. Two-species consortium

**Figure 3.**
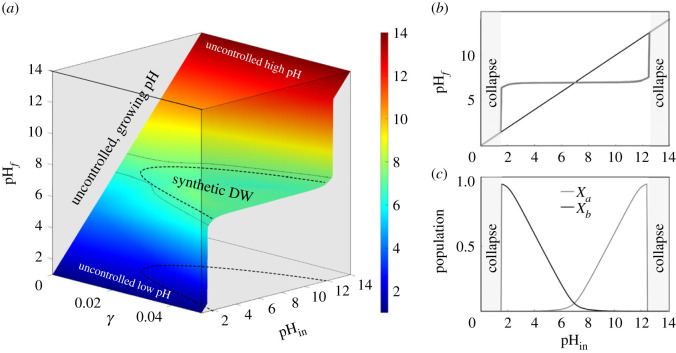
Steady state surface for the synthetic Daisyworld are depicted using equations (2.1) and (2.2). The flat surface displayed in (*a*) corresponds to the self-regulation illustrating dynamics of the synthetic microbial Daisyworld. The relationship between pH_in_ and pH_*f*_ is presented against *γ*. When *γ* = 0, neither base nor acid production occurs, resulting in the absence of regulation and a linear increase in pH_*f*_ with the external input. Conversely, as *γ* increases, a diverse range of controlled pH values emerges, expanding the homeostatic region of optimal pH. In (*b*), one section to the surface at *γ* = 0.04 is made, showing the broad domain of self-regulation surrounded by the collapse domains. The corresponding abundances of each synthetic strain are displayed in (*c*). *X*_*a*_ stands for acid-producing species and *X*_*b*_ for base-producing. Here, we used: *γ* ∈ [0, 0.05], *δ* = 0.01, *ω* = 0.5 and both species use pH_opt_ = 7 and pH_lim_ = 9.

Figure 3 illustrates the behaviour of the synthetic Daisyworld design, using the set of equations described in section §2.2 of the Methods. Here, two species with the same physiological preferences in pH but opposite effects on the surrounding pH establish a wide zone of homeostasis. Similar to the classic work of Watson & Lovelock with temperature, the key parameter (pH) also increases in the environment where both species coexist. The surface in figure 3*a* fully captures the presence and range of the homeostatic self-regulation achieved by our system. The three axes correspond to the external pH input (pH_in_), the actual pH in the media (pH_*f*_) and the *γ* parameter that gives age rates of the acid/base production. For very small *γ* values, the strains have no effect on the media pH and thus pH_*f*_ = pH_in_.

As *γ* increases, self-regulation emerges leading to a bell-shaped curve. This phenomenon can be observed more clearly in figure 3*b*, which represents a two-dimensional graph showing pH_*f*_, against pH_in_, bounded by two extreme scenarios that would lead to ecosystem collapse (grey areas). If species have no effect on the surrounding pH or there are simply no species alive, the result is a straight line representing both pH measures, therefore, producing an uncontrolled environment. When a sufficiently high production rate is reached, homeostasis around the optimum appears.

In figure 3*c*, we plot the corresponding equilibrium populations against input pH. The involved strains undergo a switch, therefore, inducing homeostasis. The base production species (*X*_*b*_) grows rapidly at low pH_in_ since it is able to thrive in acidic environments by alkalizing its surroundings. When the pH is high enough, the acidifying strain experiences a boost and out-competes the first strain until the pH becomes too high, resulting in the collapse of the system. Due to their presence, the pH is regulated around seven within an effective range 3 ≤ pH_in_ ≤ 12, allowing both species to grow within this span. These results are fully consistent with the original DWM and support our proposal that a synthetic, small-scale implementation of planetary regulation can be formulated. How robust is this result? In the next two sections, we answer this question under two distinct and relevant scenarios.

### 3.2. Two-species plus parasites

The conceptual framework of the DWM is grounded in the presence of cooperative control of the environmental fluctuations. Such control is operated, as described above, by a two-species consortium. The effective outcome of the interactions between the members of the consortium is the stabilization of both populations, although submitted to a marked bias related to the pH input value. How robust is this mechanism of environmental stabilization? One way of answering this question is to consider the introduction of a parasitic component. Parasites are known to destabilize cooperative systems, sometimes pushing the population to the extinction threshold [45–47]. Such a scenario has been tested in the context of synthetic biology, using a mixotrophic consortium growing in a 2D surface along with a parasitic strain [20].

What is the effect of an added parasitic strain? To answer this question, we consider an extended, three-species model where the new set of equations reads as

3.1 $$\frac{dX_{a}}{dt}=X_{a}[\phi (X)\beta ({\mathrm{pH}}_{a})-\delta ]$$

3.2 $$\frac{dX_{b}}{dt}=X_{b}[\phi (X)\beta ({\mathrm{pH}}_{b})-\delta ]$$

3.3 $$\frac{dX_{c}}{dt}=X_{c}[\phi (X)\beta ({\mathrm{pH}}_{c})\alpha -\delta ]$$

Here *X*_*a*_ and *X*_*b*_ are the two previously defined strains and *X*_*c*_ stands for new strain. The logistic term now reads $\phi (X)=1-(X_{a}+X_{b}+X_{c})$. A growth rate parameter *α* ∈ [0, 2] has been introduced in d*X*_*c*_/d*t* in order to tune the relative advantage of the extra species in relation to the pair of regulating species. The rest of parameters remain the same. In order to define a parasitic interaction, the functional form of the *β* term for *X*_*c*_ reads as

3.4 $$\beta ({\mathrm{pH}}_{c})=1-\frac{{\left({\mathrm{pH}}_{\mathrm{opt}}-{\mathrm{pH}}_{\;f}\right)}^{2}}{{\left({\mathrm{pH}}_{\mathrm{opt}}\pm {\mathrm{pH}}_{\lim}\right)}^{2}}$$

Therefore, as defined, the added species takes advantage of the two other strains, but (as it must be for a parasite) has no effect on the free pH and thus makes no contribution to regulation.

**Figure 4.**
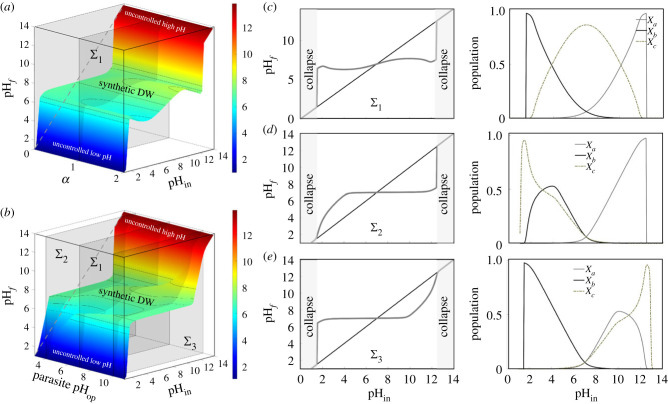
Effects of parasitism (using equations (3.1)–(3.3)) on self-regulation domains. In (*a*), the relationship between pH_in_ and pH_*f*_ is displayed against *α* as a control parameter, weighting the parasite advantage relative to the regulating species. In (*b*), the relationship between pH_in_ and pH_*f*_ is shown for parasites with pH_opt_ ∈ [3, 11] and pH_lim_ = pH_opt_ + 2.5 are introduced, with *α* = 1. The sections obtained from the vertical planes Σ cutting through both surfaces are depicted in (*c*–*e*), planes $\Sigma_{1}$, $\Sigma_{2}$ and $\Sigma_{3}$, respectively. The three cases correspond (for *α* = 1) to parasite optimal values of (*c*) pH_opt_ = 7, (*d*) pH_opt_ = 4 and (*e*) pH_opt_ = 10. The corresponding population abundances are depicted in the right column. The remaining parameters are the same as those used in figure 3.

In figure 4, we summarize the results of our theoretical model. The surface shown in figure 4*a* dramatically illustrates the fact that the parasite does not undermine the homeostatic effect; instead, it maintains it, enabling the parasite to thrive in an environment with increasing pH levels. In figure 4*a*, we clearly appreciate the presence of a very broad domain of regulation.

The section defined for the neutral case *α* = 1 (vertical, grey plane) is shown in figure 4*c* along with the corresponding populations (right column). The homeostasis region appears mildly perturbed, and remains constant with *α* (see surface figure 4*a*). The pH_in_ versus *Population* graph shows that the parasite is able to thrive almost reaching carrying capacity when all share optimal pH. What if they do not share an optimal pH? Figure 4*b* shows the robustness of the homeostatic effect. Here the parasite pH_opt_ ranges from 3 to 11 and *α* has been set to 1. Obviously, when pH_opt_ = 7 we have the same plane $\Sigma_{1}$. When the pH_opt_ is acidic or alkaline (planes $\Sigma_{2}$ and $\Sigma_{3}$, respectively), the perturbation in the homeostasis happens at those specific ranges. Plane $\Sigma_{2}$ (figure 4*b*,*d*) experiences a delay in the regulatory effect, diminishing the presence of *X*_*b*_. The inverse effect is expected in $\Sigma_{3}$ (figure 4*b*,*e*). In summary, the introduction of a parasitic component does not (at all) have a detrimental effect on self-regulation. Our model demonstrates that the properties of a pH-regulated DWM are equivalent to those that have already been described for more commonly studied thermostatic DWMs.

**Table 1.** Functions and parameters defined throughout the paper.

variable	description
*X*_*a*_, *X*_*b*_, *X*_*c*_	acid-producing, base-producing and parasite cell concentration
*ϕ*(*X*_*i*_)	function representing negative feedback due to limited space
*β*(pH_*i*_)	growth rate as a function of the perceived external pH value
pH_opt_, pH_lim_	optimal pH value and pH limit, respectively, at which cells can grow
pH_*a*_, pH_*b*_	external pH experienced by acid-producing and base-producing cells
pH_*f*_	pH in the media
pH_in_	external input pH
*a*_*e*_, *b*_*e*_	steady state external levels of acid and base produced by a cell
*a*_*i*_, *b*_*i*_	steady state internal levels of acid and base produced by a cell
*γ*	production rate of acid/base inside a cell
*k*_1_, *k*_2_, *k*_3_, *k*_4_	interchange rates between inner cell and exterior for acid and base
*δ*	dilution rate
*ω*	cell sensitivity to the produced acid/base
*α*	growth rate parameter for parasitic cells
ϵ	immigration rate for each species in the multispecies model
*b*	midpoint of the possible pH range in the multispecies model

### 3.3. Multispecies synthetic Daisyworld: order and chaos

Low-dimensional ecosystems made of two or three species provide the simplest limit cases where general principles can be derived. As it occurs with genetic switches or simple oscillatory systems, a few components coupled through a nonlinear set of interactions can display robust behaviour [48]. Here too, the two-species example is our proof of concept that planetary regulation scenarios can be properly represented at small scales. What about a multispecies scenario? Previous work on the DW model has also considered the impact of biodiversity by introducing a range of competitor species displaying different parameters, also including different trophic levels [49]. Here, we aim to explore the capacity for self-organization within a community where members have different pH_opt_ and pH_lim_ values that we generate at random, as well as their different effects on pH. The question we seek to answer is whether the community can persist while stabilizing the environmental pH. It is important to note that multi-species population dynamics under nonlinear regimes explored here are likely to develop oscillations and chaos [50]. Will homeostasis also emerge in these cases? How is it affected by species diversity?

To address these questions, a new set of equations can be used. Those describing cell populations are already familiar to us:

3.5 $$\frac{dX_{i}}{dt}=X_{i}[\phi (X)\beta ({\mathrm{pH}}_{i})-\delta ]+\epsilon$$

Here, *X*_*i*_ represents the concentration of pH-modifying bacteria, where *i* = 1, 2, …, *n*. Here $\phi (X)=1-\sum_{\;j}^{n}X_{\;j}$ is the negative feedback associated with finite resources, *δ* represents the dilution rate, and an immigration factor ϵ is introduced. The growth rate *β* has the same functional form as before, but now extended to multiple species:

3.6 $$\beta =1-\frac{1}{\Delta_{H}^{2}}{\left[{\mathrm{pH}}_{\mathrm{opt}}-{\mathrm{pH}}_{\;f}+\frac{\omega }{\delta }\sum_{i=1}^{n}\gamma_{i}\right]}^{2}$$

Here, Δ_*H*_ = pH_opt_ − pH_max_, and *γ* represents the production rate of acid or base, which can be positive or negative depending on whether it is an acid or base producer. The range of *γ* is given by *γ* ∈ ±[*γ*_min_, *γ*_max_]. The dynamics for pH_*f*_ experience a slight change and can be described as

3.7 $$\frac{{\mathrm{dpH}}_{\;f}}{dt}=\left(\sum_{i=1}^{n}X_{i}\frac{\gamma_{i}\omega }{\delta }\right)\left(\frac{{\mathrm{pH}}_{\;f}}{b}(2b-{\mathrm{pH}}_{\;f})\right)$$

Here, the expression differs from our previous models. First, there is no pH_in_, indicating that the environment does not change due to external factors; rather, the community itself is the only changing factor. Additionally, following [51], since multiple factors (species) influence the pH, a saturation term has been introduced that ensures that pH_*f*_ remains within the range of [0, 2*b*] (here *b* = 7).

As it occurs with most high-dimensional, non-spatial dynamical systems, complex dynamical states might often involve high-amplitude oscillations, hindering species from establishing a suitable environment before collapse [52,53]. To address this limitation of well-mixed models, one effective approach is the introduction of a dispersal term or immigration factor [54]. Both theoretical and empirical studies have demonstrated that moderate levels of dispersal serve as a primary mechanism for survival, disrupting high-amplitude population dynamics [54–56]. Furthermore, in challenging environments, evolutionary rescue has been observed as a means of population recovery [57]. In a mean-field model such as ours, this effect can be effectively simulated through immigration, thus serving as a proxy for both space and evolution, while maintaining the model in its minimal state [58]. All species are set in an initial pH = 7 environment.

**Figure 5.**
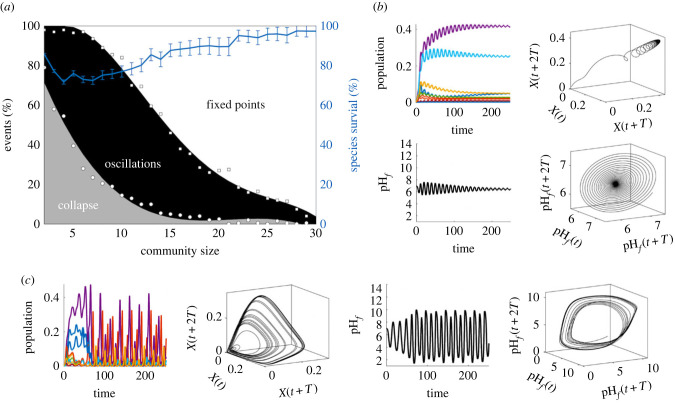
Statistical analysis and specific examples are illustrated for the multispecies case study. Three different final outcomes are obtained, as summarized in (*a*): extinction due to collapse and failure of regulation, oscillatory, quasi-periodic or chaotic fluctuations (black) and self-regulating communities displaying constant values after a transient. Although chaotic, oscillatory and collapse states are frequent at low-species numbers, they become rare as diversity increases. Raw data in squares and circles. Two examples of stable (*b*) and chaotic (*c*) communities are also shown. In both cases, we show the time series of populations, the reconstructed attractor of one of them, the pH time series and its reconstructed attractor are displayed. The population and pH dynamics for case (*c*) define strange, chaotic attractors. In addition, the mean percentage of the original species that survive for each initial community size is displayed in (*a*), in the right *y* axis (blue). Standard deviation is also plotted with error bars. Here, we used *N* = 200 replicas for each community size (up to *n* = 30 species), where parameters have been randomly generated within the intervals pH_opt_ ∈ [4, 9], pH_lim_ = pH_opt_ + [1, 4] and *γ* ∈ [0.05, 0.2], *δ* = 0.1, using *ω* = 1 and a small immigration factor of $\epsilon =10^{-3}$ has been used.

In order to characterize the general patterns of organization emerging from this high-dimensional set of equations, a statistical analysis of their dynamical behaviours has been performed, using different randomly generated communities. The analysis allows us to see the frequency of different dynamical states as a function of diversity (figure 5*a*). Following previous methods developed elsewhere [59], we use the number of species as a parameter to determine the likelihood to observe our final community in one of these three dynamical states: (i) stable fixed point attractors, (ii) oscillations and chaos and (iii) collapse. These correspond to communities achieving populations that remain constant over time (after some transient), populations that exhibit deterministic fluctuations (either periodic, quasi-periodic or chaotic) and finally those that experience extinction after a short initial instability. In figure 5*a*, we employed a polynomial fitting of degree 4 for circles (raw data) and degree 3 for squares (raw data) to enhance the visualization of the dynamical states. Additionally, (figure 5*a*, right axis), we show the fraction of the original species that survive for each initial community size.

For each sampled model ecosystem, we discard a long transient to avoid non-stationary effects. Oscillatory or chaotic behaviours are found by looking at the amplitude variations of the time series of any of the species involved, and we do not distinguish among the different kinds of dynamics. Examples of both point attractors and fluctuating populations, along with the corresponding pH_*f*_ time series are displayed in figure 5*b*,*c*, respectively. We also display three-dimensional reconstructions of the underlying attractors. These are obtained by plotting the dynamics of one chosen species with a population *X*(*t*) on a delayed three-dimensional space using the vectors *X*(*t*), *x*(*t* + *T*), *x*(*t* + 2*T*) using a time delay *T* [60,61].

Interestingly, the observed fractions of dynamical states as a function of diversity allow us to formulate a strong prediction: despite the dominant presence of fluctuating populations for high-dimensional ecosystems, increasing biodiversity has a suppressor effect, favouring point attractors. In other words, species diversity enhances the desired self-regulation effects. It is also worth mentioning that earlier higher diversity seems to increase the chances of a stable sub-community surviving an early upheaval period and going on to regulate their environment due to their complementary environmental impacts, as can be seen in figure 5*a*. This is in line with previous models on planetary self-regulation that have shown how community restructuring can lead to stability [62].

**Figure 6.**
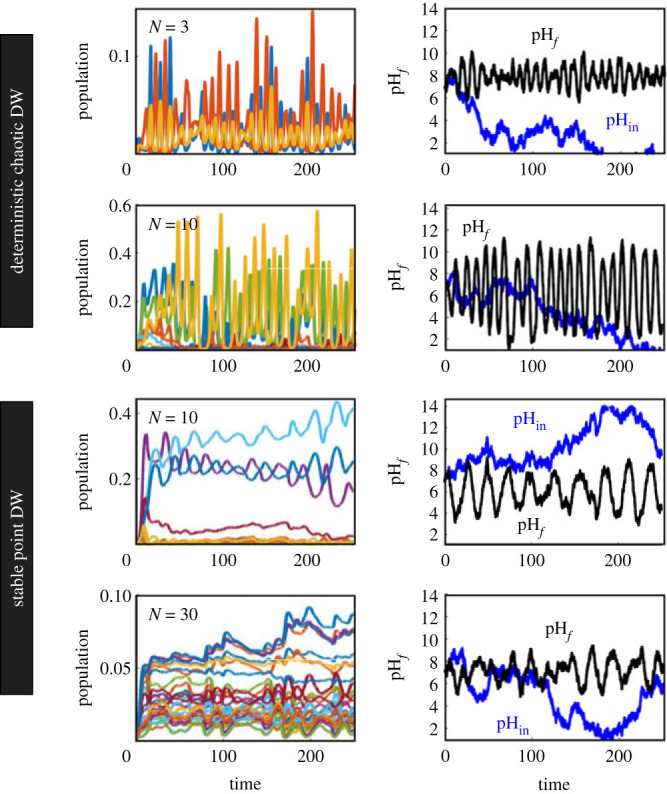
Homeostatic behaviour for multispecies models under stochastic variations in the pH input. Here, we have chosen for different conditions associated with both stable (point attractors) and oscillating communities (as defined from the phase space in figure 5*a*). Specifically, the cummulative input pH_in_ fluctuates in time (blue lines) starting from pH_in_(0) = 7. The time series for populations (left) and pH values (right) are shown. All communities respond by adjusting pH_*f*_ (black lines) which typically fluctuate within abounded range of values around the deterministic regulated value.

Is this homeostasis preserved when exogenous fluctuations of pH are present? Will fluctuating populations (such as those in the ‘oscillations’ phase of figure 5) cope with a time-dependent, noisy input? We have explored this problem by introducing a stochastic component in the time evolution of pH_in_. The multispecies synthetic Daisyworld not only self-organizes to configure its environment optimally, but also exhibits the capacity to buffer external pH perturbations, sustaining oscillatory homeostasis in the face of a fluctuating environment. The external perturbation takes the form of an additive (discrete) uniform white noise sampled at every time step, characterized by a zero mean, and magnitudes between [−5, 5] over an interval. In figure 6, we show several examples of these responses for four different community sizes associated with either chaotic or stable point attractors, respectively. In all these cases, it is found that the community responses display pH buffering and thus robust self-regulation. Further work should address the statistical patterns displayed by stochastic versions of our previous models.

## 4. Discussion

The DWM provides a rationale for a stable self-organized biosphere resulting from a feedback between living beings and their environment, which they modify in predictable ways. Despite (or perhaps because of) its simplicity, the DWM has been instrumental in developing the field of Earth systems science [13]. Can new synthetic ecosystems help further develop the field by allowing experimental testing in the test tube? Previous work on synthetic ecosystems, both *in vivo* and *in silico*, have considered communities that illustrate the success or failure of feedback control in microbial ecosystems. Examples include ecosystem-level nutrient recycling in the *Flask* model [63] or the potential for collapse (ecological suicide) in microbial communities where sustained modification of environmental pH by the microbes can end in their extinction [64]. Our proposal instead is that of an engineered biological system capable of self-adjusting itself under a given range of external parametric conditions, actively acting on stabilizing a global environmental driver. We propose a specific design for the genetic constructs required to self-tune the system. The one-humped nature of growth responses against pH displayed by microorganisms makes our candidate designs perfectly fit to match the original DWM assumptions. Moreover, under a multispecies context, biodiversity is a firewall to prevent the system from becoming de-regulated. This, in our view, represents a novel concept.

There are several potential extensions of our work that are worth exploring. On one hand, the regulatory nature of the system described here can be extended to other systems beyond the ecological context considered here. One avenue is the potential of terraformation scenarios based on synthetic biology, where cooperative consortia might be required for a successful spread over large scales [65]. Another one concerns those physiological systems (such as glucose regulation by glucagon and insulin) that can be described in similar terms [66,67]. These ‘rein control’ systems could be constructed synthetically and used to target given regulation goals within a model organism. Finally, we have not included a major player in Lovelock’s picture: evolutionary dynamics. Previous work on evolutionary dynamics in DWMs [68–71]. The role played by evolution has been a matter of intense discussion within the context of the DWM [3,72] and we may wonder if some of these debates could be settle by evolving synthetic communities. Extensions of our model approach could guide future developments towards this goal.

## Data Availability

The code used for the simulations is available from the Zenodo repository: https://doi.org/10.5281/zenodo.10475383 [73].
